# Antinociceptive and Anti-Inflammatory Effects of Recombinant Crotamine in Mouse Models of Pain

**DOI:** 10.3390/toxins13100707

**Published:** 2021-10-06

**Authors:** Jong Yeon Park, Bich Hang Do, Ju-Seung Lee, Hyun Cheol Yang, Anh Ngoc Nguyen, Martin Krupa, Chong Jai Kim, Yeon Jin Jang, Han Choe

**Affiliations:** 1 Department of Anesthesiology and Pain Medicine, Asan Medical Center, University of Ulsan College of Medicine, Seoul 05505, Korea; christa416@naver.com (J.-S.L.); sajangxyz@daum.net (H.C.Y.); 2 Faculty of Pharmacy, Ton Duc Thang University, Ho Chi Minh City 70000, Vietnam; dobichhang@tdtu.edu.vn; 3 Department of Physiology, Biomedical Institute of Technology, Asan Medical Center, University of Ulsan College of Medicine, Seoul 05505, Korea; ngocnabio@gmail.com (A.N.N.); martinkrupa@gmail.com (M.K.); yjjang@amc.seoul.kr (Y.J.J.); 4 Department of Pathology, Asan-Minnesota Institute for Innovating Transplantation, Asan Medical Center, University of Ulsan College of Medicine, Seoul 05505, Korea; ckim@amc.seoul.kr

**Keywords:** crotamine, nociception, inflammation, naloxone, tumor necrosis factor-α

## Abstract

Crotamine, a toxin found in the venom of the South American rattlesnake *Crotalus durissus terrificus*, has been reported to have antinociceptive effects. We purified recombinant crotamine expressed in *Escherichia coli* and investigated its antinociceptive and anti-inflammatory effects using the hot-plate test, acetic-acid-induced writhing method, and formalin test in mice. Recombinant crotamine was administered intraperitoneally (0.04–1.2 mg kg^−1^) or intraplantarly (0.9–7.5 μg 10 μL^−1^) before the tests. The paw volume was measured with a plethysmometer. To evaluate the antagonistic and anti-inflammatory effects of naloxone, subcutaneous naloxone (4 mg kg^−1^) or intraplantar naloxone (5 μg 10 μL^−1^) was administered before recombinant crotamine. For tumor necrosis factor (TNF)-α assays, blood was drawn 3 h after formalin injection and measured using enzyme-linked immunosorbent assay. Intraperitoneal and intraplantar recombinant crotamine had antinociceptive and anti-inflammatory effects, neither of which were affected by pre-treatment with naloxone. The mean serum TNF-α levels were significantly lower in the intraperitoneal recombinant crotamine (0.4 and 1.2 mg kg^−1^) or intraplantar (2.5 and 7.5 μg 10 μL^−1^) recombinant crotamine groups than in the saline group and were not affected by naloxone pre-treatment. In conclusion, recombinant crotamine possesses significant antinociceptive and anti-inflammatory effects that do not appear to be related to the opioid receptor. The antinociceptive and anti-inflammatory effects of intraperitoneal or intraplantar recombinant crotamine are related to TNF-α.

## 1. Introduction

The antinociceptive properties of snake venoms have been well known [1,2]. For example, cobrotoxin, a neurotoxin found in the venom of *Naja atra* (Chinese cobra) and other cobras, has strong analgesic effects in animal models [1,2]. These effects were not affected by naloxone, an antagonist of opioid receptors, and were more potent than opioids. A bite of the most lethal South American snake, *Crotalus durissus terrificus*, was also reported to have analgesic effects [3]. The venom of the rattlesnake is a mixture of at least 67 proteins and small peptides [4,5]. The most abundant protein in the venom is crotamine of 4.9 kDa [6,7]. There has been a report on crotamine’s antinociceptive activity [8]. When injected in mice, native crotamine purified from the snake venom exerted time- and dose-dependent analgesic effects. Compared with morphine, the native crotamine was approximately 500-fold more potent on a molar basis. Interestingly, unlike the cobrotoxin, the native crotamine’s analgesic effects were inhibited by naloxone, suggesting the involvement of the opioid receptors [8]. Another protein smaller than 3 kDa from the snake venom also showed analgesic effect that was inhibited by naloxone [9].

Due to its powerful analgesic activity, crotamine is a good candidate for an anti-pain drug, and development would require efficient production of recombinant crotamine on a large scale. Eukaryotic heterologous production of recombinant crotamine was difficult because the protein was both cell-permeable and cytotoxic. Prokaryotic expression of recombinant crotamine using *Escherichia coli* (*E. coli*) resulted in insoluble fraction and showed circular dichroism spectra different from that of the native crotamine, suggesting that recombinant crotamine was misfolded [10]. We solved this misfolding problem by genetically fusing maltose binding protein (MBP) as a chaperone tag to the N-terminus of the crotamine gene and documented in the report [11]. The fusion protein was expressed as a soluble form suggesting a correct folding of the recombinant protein. We purified the fusion protein and removed the MBP tag. The final highly-pure crotamine was shown to have correct disulfide bonds based on MALDI-TOF mass analysis [11]. Our homemade recombinant crotamine also inhibited the voltage-gated potassium channel, hKv1.3, but not the closely related channel, hKv1.5 [11], which is consistent with the result obtained using the native crotamine [12]. The IC_50_ of recombinant crotamine was 67 nM [11], which is lower than 300 nM of the native crotamine [12], suggesting the correct folding and also a higher purity of recombinant crotamine.

In this study, we investigated the antinociceptive and anti-inflammatory effects of our homemade recombinant crotamine produced from *E. coli* [11] using behavioral tests. We also investigated serum tumor necrosis factor (TNF)-α levels as a surrogate biochemical marker of inflammation. Interestingly, the antinociceptive effects of the homemade recombinant crotamine were not antagonized by naloxone, which was similar to cobrotoxin [1,2] and different from the previous report of crotamine [8].

## 2. Methods

### 2.1. Expression and Purification of Recombinant Crotamine from E. coli

In this study, only recombinant crotamine from *E. coli* produced in our lab but not the native crotamine from the snake venom was used. The molecular cloning, expression, purification, and characterization of the purified crotamine were described in detail in our previous report [11]. In this study, the recombinant protein was prepared using the described method with the following modifications. A HisTrap Fast Flow affinity column (GE Healthcare, Piscataway, NJ, USA) was used to purify the fusion MBP-crotamine protein. After TEV treatment, the sample was applied to a 5 mL HiTrap SP HP column (GE Healthcare) and equilibrated with buffer containing 20 mM Tris-HCl, 5% glycerol [*v*/*v*], pH 8.0. The cleaved crotamine was eluted with 2 M NaCl after washing with 100 mM NaCl. The gel filtration column (GE Healthcare) was equilibrated with buffer containing 20 mM Tris-HCl, 500 mM NaCl, 5% glycerol [*v*/*v*], pH 8.0, and applied as the final column to remove any remaining contaminants. The purified crotamine was exchanged with phosphate-buffered saline (PBS) using dialysis. Protein concentrations were measured using the bicinchoninic-acid assay (Pierce Biotechnology, Rockford, IL, USA). The protein was stored at −80 °C until use.

### 2.2. Experimental Animals

Six-week old male ICR mice (25–30 g, Orient, Seoul, Korea) were used in all experiments. Animals were housed 4–5 per cage in a room maintained at 22 ± 0.5 °C with an alternating 12 h light–dark cycle. Food and water were available *ad libitum*. The animals were allowed to adapt to the laboratory environment for at least 2 h before testing. Experiments were performed during the light phase of the cycle (10 a.m.–5 p.m.). 

### 2.3. Behavioral Tests for Antinociceptive Activity Assay of Recombinant Crotamine

#### 2.3.1. The Hot-Plate Test

Each group (*n* = 7) of mice was placed on a hot-plate (55 ± 1 °C, Ugo Basile, Comerio-Varese, Italy) and confined inside a transparent acrylic cylinder on the plate. Response latency (baseline), the time until the first response to the heat stimulus (licking of either front or back paws), was then measured. After 30 s, 100 μL of recombinant crotamine was intraperitoneally injected. Recombinant crotamine doses were 0.04, 0.13, 0.4 and 1.2 mg kg^−1^ (Table 1). Control animals received only saline (100 μL). Response latency was measured again after 20, 40, 60, 80, and 100 min. As the same animal was submitted to several measures at 20 min intervals, the cut-off value (maximal length of time on the hot-plate) was limited to 30 s to avoid any injury to the mouse paw.

To evaluate the antagonistic effect of naloxone, 100 μL of naloxone (4 mg kg^−1^) was subcutaneously administered 10 min [8] before the intraperitoneal injection of the same recombinant crotamine or saline doses as above. Naloxone has an onset of action within 1–2 min following IV administration and within 2–5 min following subcutaneous or IM administration [13].

#### 2.3.2. The Acetic-Acid-Induced Writhing Method

Each group (*n* = 7) of mice received the same doses of recombinant crotamine (0.04, 0.13, 0.4, and 1.2 mg kg^−1^) intraperitoneally (Table 2). Control animals received only saline (100 μL). After 20 min [14], 100 μL of a 0.6% (*v*/*v*) acetic-acid solution was intraperitoneally administered, and mice were immediately returned to the transparent acrylic observation cage (Supplementary Figure S1). The number of writhes was then measured for 20 min [15]. To evaluate antagonistic effects of naloxone, 100 μL of naloxone (4 mg kg^−1^) was subcutaneously administered 10 min [8] before the intraperitoneal injection of the same recombinant crotamine doses or saline as above. Naloxone works to reverse opioid overdose in the body for only 30 to 90 min [16].

#### 2.3.3. The Formalin Test: Systemic (Intraperitoneal) Effect of Recombinant Crotamine

Each group (*n* = 7) of mice received intraperitoneal doses of recombinant crotamine (0.13, 0.4, and 1.2 mg kg^−1^) (Table 3). Negative-control animals received only saline (100 μL), and positive-control animals received indomethacin (10 mg kg^−1^). After 20 min, the formalin test was performed, in which, 10 μL of a 2.5% formalin solution was injected subcutaneously into the plantar surface of the left hind paw with a 30 G needle. The mice were immediately returned to the transparent acrylic observation cage and placed over a mirror angled at 40–50° to allow for an unobstructed view of the paws.

A formalin-induced pain test is usually divided into two phases; phase 1 lasts about 10 min and phase 2 lasts about 50 min [17,18]. In phase 2, the peak mean nociceptive score is produced 20–30 min after formalin injection, and mean nociceptive score is gradually decreased after that time. The mean nociceptive score at the 60 min time point is usually 0–0.5. Therefore, we observed the nocifensive behaviors for the next 60 min. The formalin-induced pain was divided into phases 1 (0–9 min) and 2 (10–60 min). Four behavioral categories were devised to quantify pain: 0, left hind paw corresponds with right hind paw and is used normally by the mouse; 1, left hind paw has little or no weight laid on it; 2, left hind paw is lifted in the air and is not in contact with the bottom or lateral wall of the observation cage, and; 3, left hind paw is flinched, licked, or bitten. The recording period was divided into 3 min blocks. The mean nociceptive score for each block was determined by measuring the time spent in each of the four behavioral categories. The weighted nociceptive score ranged from 0 to 3; it was calculated by multiplying the time (in s) used for each category with its assigned category weight. These summated products were divided by the total time for each 3 min (=180 s) block of time. The formula is summarized as follows: mean nociceptive score = (1T1 + 2T2 + 3T3)/180. T1, T2, and T3 indicate the time (in s) spent in categories 1, 2, and 3, respectively. The total area under the curve (AUC) was also calculated for each mouse [19].

To evaluate the antagonistic effect of naloxone, 100 μL of naloxone (4 mg kg^−1^) was subcutaneously administered 10 min [8] before the intraperitoneal injection of recombinant crotamine (0.4 and 1.2 mg kg^−1^) or saline.

#### 2.3.4. The Formalin Test: Peripheral (Intraplantar) Effect of Recombinant Crotamine

Each group (*n* = 7) of mice received intraplantar administration of saline (10 μL) to the left hind paw with a 30 G needle. After 15 min, recombinant crotamine (0.9, 2.5, and 7.5 μg 10 μL^−1^) (Table 4) was injected subcutaneously into the plantar surface of the left hind paw with a 30 G needle. Negative-control animals received only saline (10 μL), and positive-control animals received indomethacin (50 μg 10 μL^−1^) instead of recombinant crotamine. After 15 min, the formalin test was performed in the same manner as described above.

To evaluate antagonistic effects of naloxone, naloxone (5 μg 10 μL^−1^) was subcutaneously administered into the plantar surface of the left hind paw 15 min before the intraplantar injection of recombinant crotamine (2.5 and 7.5 μg 10 μL^−1^) or saline. There was no reference for how long naloxone should be treated in a mouse paw. Therefore, we waited 5 min longer than subcutaneous administration (10 min) to make sure sufficient diffusion.

### 2.4. Behavioral Tests for Anti-Inflammatory Activity Assay: Systemic (Intraperitoneal) and Peripheral (Intraplantar) Effect of Recombinant Crotamine in Formalin Test

The swelling of the left hind paw was measured as described previously [20]. The left hind paw of a mouse was submerged to the ankle hairline within a plethysmometer (Ugo Basile, Italy), measuring the paw volume (mL). In general, formalin-induced paw edema gradually increases and reaches the maximal values 3 days after injection of formalin [21]. We also measured the serum TNF-α. The serum TNF-α is usually measured 1–6 h after administration of some stimulants to verify the acute effects [22]. Therefore, the measurements of the paw volume (mL) were performed before and 3 h after an intraplantar injection of formalin into the left hind paw. The paw swelling (%) was calculated using the formula: paw swelling (%) = (paw volume value 3 h after formalin administration - paw volume value before the injection) × 100/(paw volume value before the injection).

### 2.5. Biochemical Assays

After 3 h following the formalin injection, blood was drawn from the heart under sevoflurane anesthesia for serum analysis. For TNF-α assays, serum was separated from the blood samples by centrifugation at 4000 rpm for 30 min at 4 °C. These samples were preserved at −80 °C until use. The serum TNF-α levels were measured by a commercially available enzyme-linked-immunosorbent assay (ELISA) kit (R&D Systems, Minneapolis, MN, USA; sensitivity: 5 pg mL^−1^). The assay kit was used according to the manufacturer’s recommendations. The Bradford protein assay was used to measure the total protein concentration in the serum. ELISA microplates were analyzed using a Tecan Sunrise Microplate Reader (Salzburg, Austria) at 450 nm; data were standardized as picograms of TNF-α per 200 μg of total serum protein. The TNF-α concentration was determined based on an appropriate set of internal standard curves created using recombinant mouse cytokines.

### 2.6. Behavioral Analysis: The Rotarod Test

Motor ability and coordination were evaluated using a rotarod apparatus (Ugo Basile) for mice (length, 25 cm; diameter, 4 cm) [23]. After 30 min following the intraperitoneal injection of saline or recombinant crotamine (0.04, 0.13, 0.4, and 1.2 mg kg^−1^), mice were placed on the rod while it rotated at 4 rpm. The rotation speed was increased from 4 to 40 rpm over 5 min. The latency required for the mouse to fall from the rod expressed in s, was recorded for each animal. Mice were acclimatized to the acceleration by an initial series of three training runs. The motor-coordination performance of each mouse was determined by measuring the average time for the fourth and fifth training runs. A 15 min resting period was allowed between two successive training runs.

### 2.7. Data and Statistical Analysis

All behavioral data are expressed as means ± SEM. Statistical analyses were performed using one-way analysis of variance (ANOVA) and the Kruskal–Wallis one-way ANOVA on ranks compared with the control group at the same time point between groups, followed by a Tukey’s post hoc test for multiple comparisons. The significance of the differences in the responses of treatment groups compared with the preoperative baseline value of each group was determined using one-way repeated-measures ANOVA, followed by a Tukey’s post hoc test for multiple comparisons. Statistical evaluation was performed with SigmaPlot Version 11 (Systat Software Inc., San Jose, CA, USA). *p* < 0.05 was considered statistically significant.

## 3. Results

### 3.1. The Antinociceptive Effects of Systemic (Intraperitoneal) Recombinant Crotamine: The Hot-Plate Test

**Figure 1:**
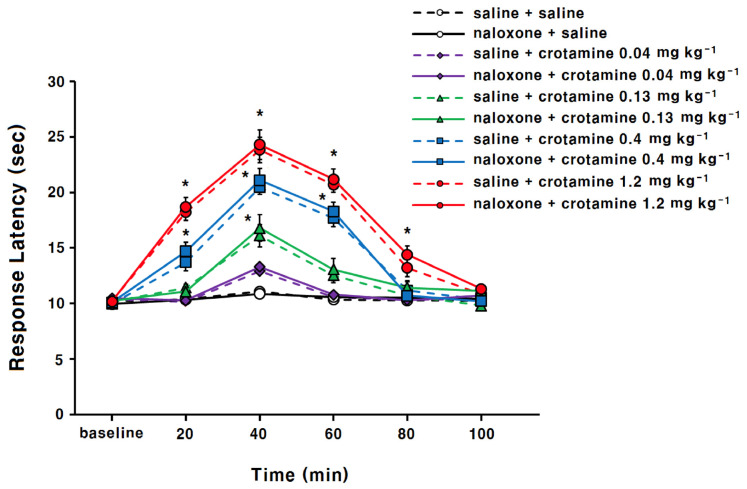
Hot-plate test of the antinociceptive activity of intraperitoneally administered recombinant crotamine in mice (*n* = 7). Naloxone (4 mg kg^−1^) was subcutaneously administered 10 min before recombinant crotamine. Full and discontinued lines denote the effects in the presence and absence of naloxone, respectively. Each value indicates mean ± SEM. * *p* < 0.001 vs. saline-control group, from one-way ANOVA with Tukey’s post hoc test.

As shown in Figure 1, the saline-control animals group had a response latency of 10.2 ± 0.2, 10.4 ± 0.3, 11.1 ± 0.3, 10.3 ± 0.3, 10.3 ± 0.2, and 10.2 ± 0.2 at the 0 (baseline), 20, 40, 60, 80, and 100 min time points, respectively. Previously, native crotamine from the snake venom showed antinociceptive activities at the concentrations of 15.6, 44.5, and 133.4 μg/kg [8]. In our pilot study, the recombinant crotamine showed no activity at 15 μg/kg (data not shown). Therefore, we selected higher doses of recombinant crotamine, 0.4 mg/kg (=400 μg/kg), and 1.2 mg/kg (1200 μg/kg) in addition to the doses of 0.04 mg/kg (=40 μg/kg), and 0.13 mg/kg (=130 μg/kg). In the recombinant-crotamine pre-treatment (0.04, 0.13, 0.4, and 1.2 mg kg^−1^) group, response latency gradually increased and peaked at 40 min (12.9 ± 0.31, 16.1 ± 0.72, 20.5 ± 0.65, 23.8 ± 0.86 s, respectively) and then progressively decreased to the baseline level at 100 min. Recombinant-crotamine pre-treatment (0.13, 0.4, and 1.2 mg kg^−1^) showed significant and dose-dependent antinociceptive effects compared with the saline-control group. In the 0.13 mg kg^−1^ recombinant-crotamine group, response latency was significantly increased at 40 min. In the 0.4 mg kg^−1^ recombinant-crotamine group, response latency was significantly increased at 20, 40, and 60 min. Moreover, in the 1.2 mg kg^−1^ recombinant-crotamine group, response latency was significantly increased at 20, 40, 60, and 80 min points.

The naloxone itself did not demonstrate any antinociceptive effects (Figure 1), and antinociceptive effects of recombinant crotamine (0.4 and 1.2 mg kg^−1^) were not affected by naloxone pre-treatment (Figure 1).

### 3.2. The Antinociceptive Effects of Systemic (Intraperitoneal) Recombinant Crotamine: Acetic-Acid-Induced Writhing Method

**Figure 2:**
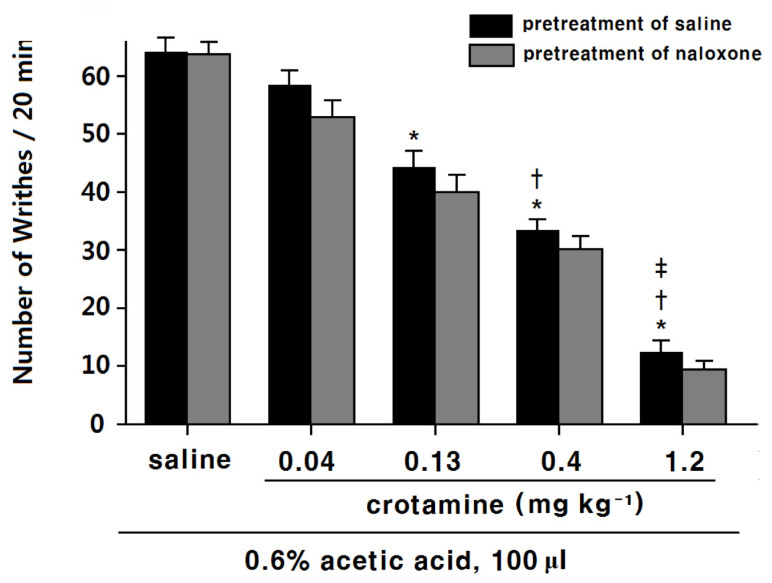
Acetic-acid-induced writhing method for the evaluation of the antinociceptive effect of intraperitoneally administered recombinant crotamine (*n* = 7). Naloxone (4 mg kg^−1^) was subcutaneously administered 10 min before recombinant crotamine. Black columns denote the absence, and gray columns the presence of naloxone. Each value indicates mean ± SEM. * *p* < 0.001 vs. saline-control group; † *p* < 0.001 vs. recombinant-crotamine (0.13 mg kg^−1^) group; ‡ *p* < 0.001 vs. recombinant-crotamine (0. 4 mg kg^−1^) group, from one-way ANOVA with Tukey’s post hoc test.

Intraperitoneally administered acetic acid induced writhing behaviors for 20 min (Figure 2). In the saline control animal group, the number of writhes was 64.1 ± 2.3. The pre-treatment of recombinant crotamine (0.13, 0.4, and 1.2 mg kg^−1^) 20 min before the acetic-acid injection showed significant and dose-dependent antinociceptive effects on the writhing response (44.3 ± 2.7, 33.4 ± 1.8, and 12.4 ± 1.9, respectively) compared with the saline-control group. In the lowest recombinant-crotamine group (0.04 mg kg^−1^), the number of writhes decreased to 58.4 ± 2.3. However, this was not significantly different from the saline-control group.

Naloxone itself did not present any antinociceptive effect (Figure 2); antinociceptive effects of recombinant crotamine (0.13, 0.4, and 1.2 mg kg^−1^) were not affected by naloxone pre-treatment (Figure 2).

### 3.3. The Antinociceptive Effects of Systemic (Intraperitoneal) Recombinant Crotamine: The Formalin Test

**Figure 3:**
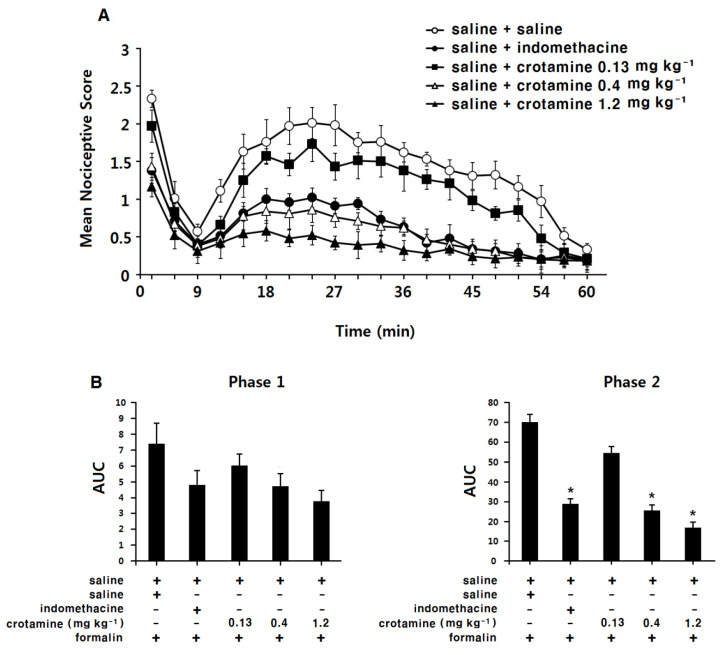
The antinociceptive effects of systemic (intraperitoneal) recombinant crotamine: the formalin test. (**A**) Time course of pain behaviors after formalin injection (*n* = 7). Indomethacin (10 mg kg^−1^) and recombinant crotamine (0.13–1.2 mg kg^−1^) were intraperitoneally pre-treated 20 min before formalin injection. (**B**) Area under the curve of pain behaviors during phase 1 (0–9 min) and phase 2 (10–60 min). Each value indicates mean ± SEM. * *p* < 0.001 vs. saline-control group, from one-way ANOVA with Tukey’s post hoc test.

Saline-control-group animals exhibited a typical biphasic nociceptive response after formalin injection into the left paw (Figure 3A). The area under the curve (AUC) was 7.4 ± 1.3 for phase 1, and 70.1 ± 3.7 for phase 2 (Figure 3B). In the indomethacin (intraperitoneal, 10 mg kg^−1^) group, the nociceptive response was significantly suppressed in phase 2, but not phase 1. The AUC was 4.8 ± 0.9 for phase 1 and 28.9 ± 2.5 for phase 2 (Figure 3B, *p* < 0.05). In the low-dose recombinant-crotamine (0.13 mg kg^−1^) group, animals showed similar nociceptive response and AUC (6.0 ± 0.7 for phase 1 and 54.4 ± 3.2 for phase 2) compared with the saline-control group. In contrast, in the high-dose recombinant-crotamine (0.4 and 1.2 mg kg^−1^) groups, intraperitoneal recombinant crotamine caused a dose-dependent suppression of the nociceptive response during phase 2, but not phase 1, of the formalin test. The AUC values were 4.8 ± 0.8 and 3.8 ± 0.7 for phase 1 and 25.6 ± 2.8 and 17.0 ± 2.6 for phase 2 (*p* < 0.05, Figure 3B). The anti-nociceptive effect of intraperitoneal recombinant crotamine (0.4 mg kg^−1^) was similar to the effect of intraperitoneal indomethacin (10 mg kg^−1^, Figure 3B).

In phase 1, there were no significant differences in AUC between each group. However, the mean nociceptive scores at the first 3 min time point produced significant differences between groups. In the low-dose recombinant-crotamine (0.13 mg kg^−1^) group, the mean nociceptive score was 2.0 ± 0.2 and it was similar to that of the saline-control group (2.3 ± 0.1). In contrast, in the high-dose recombinant-crotamine (0.4 and 1.2 mg kg^−1^) groups, the mean nociceptive scores were 1.4 ± 0.2 and 1.1 ± 0.1, respectively, and the scores were significantly suppressed compared to the saline-control group (2.3 ± 0.1). The mean nociceptive scores of the intraperitoneal recombinant-crotamine (0.4 mg kg^−1^) group (1.4 ± 0.2) was similar to the score of intraperitoneal indomethacin (10 mg kg^−1^) group (1.4 ± 0.2).

**Figure 4:**
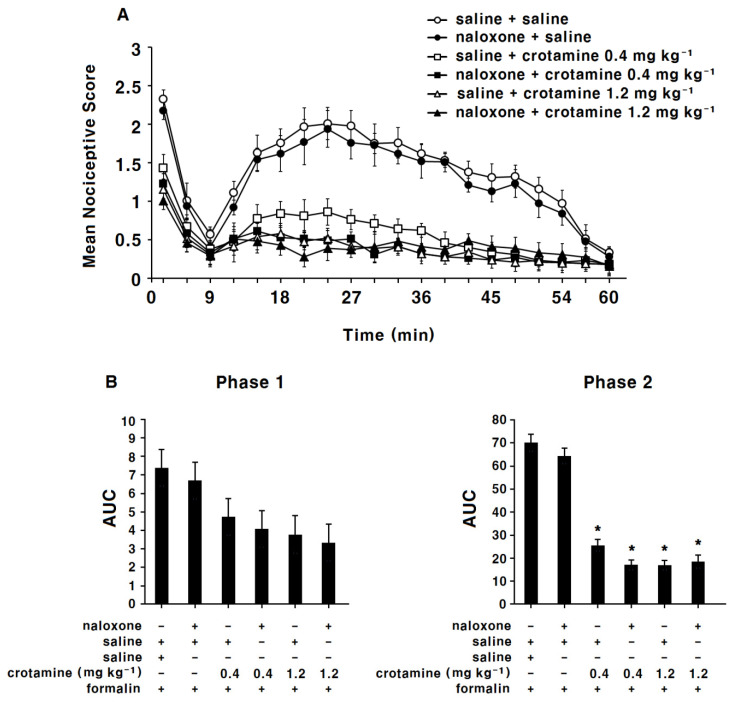
The antinociceptive effects of systemic (intraperitoneal) recombinant crotamine: pre-treatment of naloxone before the formalin test. (**A**) Time course of pain behaviors after formalin injection (*n* = 7). Naloxone (4 mg kg^−1^) was subcutaneously pre-treated 10 min before recombinant-crotamine (0.4 and 1.2 mg kg^−1^) administration. (**B**) Area under the curve of pain behaviors during phase 1 (0–9 min) and phase 2 (10–60 min). Each value indicates mean ± SEM. * *p* < 0.001 vs. saline-control group, from one-way ANOVA with Tukey’s post hoc test.

Naloxone itself did not present any antinociceptive effects (Figure 3A,B); antinociceptive effects of recombinant crotamine (0.4 and 1.2 mg kg^−1^) were not affected by the pre-treatment of naloxone (Figure 4A,B).

### 3.4. The Antinociceptive Effects of Peripheral (Intraplantar) Recombinant Crotamine: The Formalin Test

**Figure 5:**
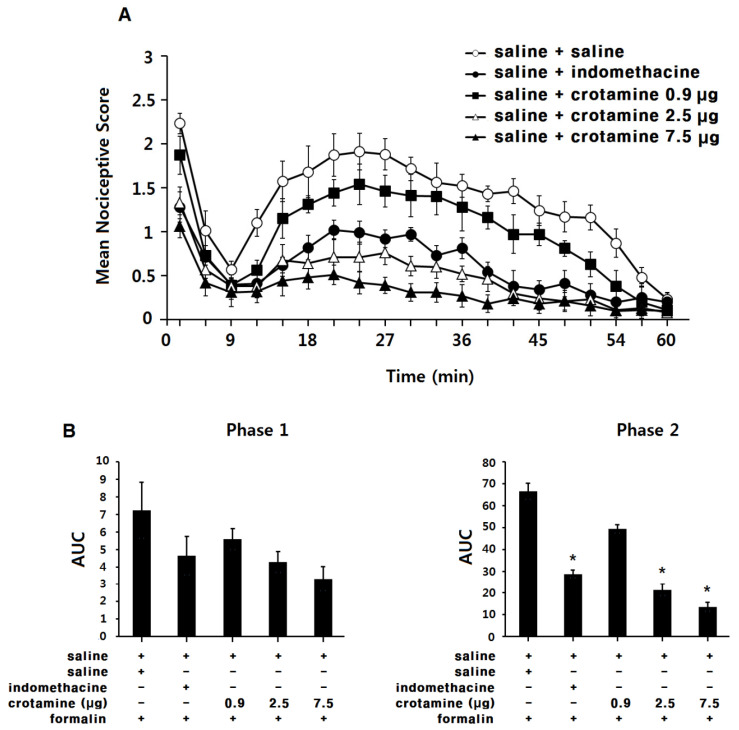
The antinociceptive effects of peripheral (intraplantar) recombinant crotamine: the formalin test. (**A**) Time course of pain behaviors after formalin injection (*n* = 7). Indomethacin (50 μg 10 μL^−1^) and recombinant crotamine (0.9–7.5 μg 10 μL^−1^) were intraplantarly pre-treated 15 min before formalin injection. (**B**) Area under the curve of pain behaviors during phase 1 (0–9 min) and phase 2 (10–60 min). Each value indicates mean ± SEM. * *p* < 0.001 vs. saline-control group, from one-way ANOVA with Tukey’s post hoc test.

Saline control animals exhibited a typical biphasic nociceptive response after formalin injection into the left paw (Figure 5A). The AUC was 7.2 ± 1.6 for phase 1 and 66.5 ± 3.7 for phase 2 (Figure 5B). In the indomethacin (intraplantar 50 μg 10 μL^−1^) group, the nociceptive response was significantly suppressed in phase 2 but not phase 1. The AUC was 4.7 ± 1.1 for phase 1 and 28.8 ± 1.8 for phase 2 (Figure 5B, *p* < 0.05). In the low-dose recombinant-crotamine (0.9 μg 10 μL^−1^) group, animals showed similar nociceptive response and AUC (5.6 ± 0.6 for phase 1 and 49.3 ± 1.9 for phase 2) compared with the saline-control group. Contrastingly, in the high-dose recombinant-crotamine (2.5 and 7.5 μg 10 μL^−1^) groups, intraplantar recombinant crotamine caused a dose-dependent suppression of the nociceptive response during phase 2, but not phase 1, of the formalin test. The AUC was 4.3 ± 0.6 and 3.3 ± 0.7 for phase 1, and 21.4 ± 2.6 and 13.6 ± 2.0 for phase 2 (*p* < 0.05, Figure 5B). The anti-nociceptive effect of intraplantar recombinant crotamine (2.5 μg 10 μL^−1^) was similar to the effect of intraplantar indomethacin (50 μg 10 μL^−1^) (Figure 5A,B).

In phase 1, there were no significant differences in AUC between each group. However, the mean nociceptive scores at the first 3 min time point produced significant differences between groups. In the low-dose recombinant-crotamine (0.9 μg) group, the mean nociceptive score was 1.9 ± 0.2 and it was similar to that of the saline-control group (2.2 ± 0.1). In contrast, in the high-dose recombinant-crotamine (2.5 and 7.5 μg) groups, the mean nociceptive scores were 1.3 ± 0.2 and 1.1 ± 0.1, respectively, and the scores were significantly suppressed compared to the saline-control group (2.2 ± 0.1). The mean nociceptive score of the intraperitoneal recombinant-crotamine (2.5 μg) group (1.3 ± 0.2) was similar to the score of intraperitoneal indomethacin (50 μg 10 μL^−1^) group (1.3 ± 0.2).

**Figure 6:**
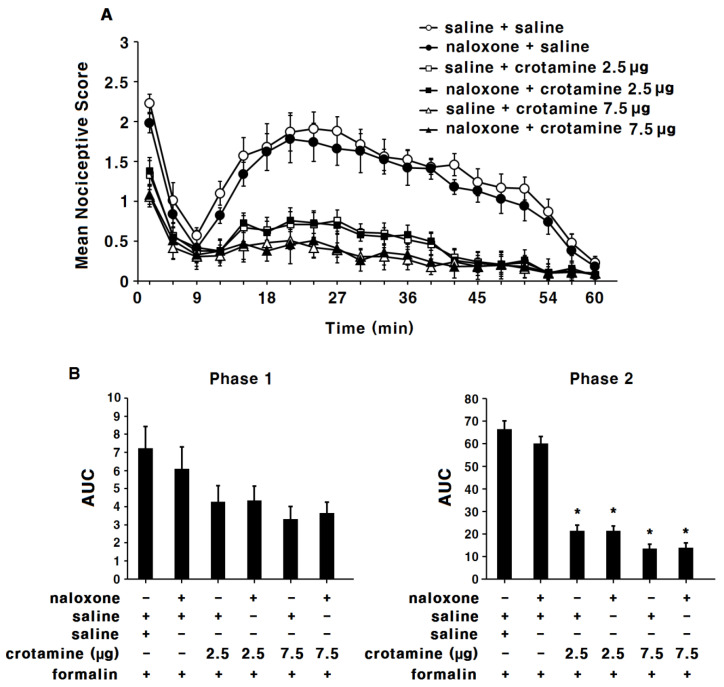
The antinociceptive effects of peripheral (intraplantar) recombinant crotamine: pre-treatment of naloxone before the formalin test. (**A**) Time course of pain behaviors after formalin injection (*n* = 7). Naloxone (5 μg 10 μL^−1^) was intraplantarly pre-treated 15 min before recombinant crotamine (2.5 and 7.5 μg 10 μL^−1^) administration. (**B**) Area under the curve of pain behaviors during the phase 1 (0–9 min) and the phase 2 (10–60 min). Each value indicates mean ± SEM. * *p* < 0.001 vs. saline-control group, from one-way ANOVA with Tukey’s post hoc test.

Naloxone itself did not present any antinociceptive effects (Figure 5A,B); antinociceptive effects of recombinant crotamine (2.5 and 7.5 μg 10 μL^−1^) were not affected by naloxone pre-treatment (Figure 6A,B).

### 3.5. The Anti-Inflammatory Effects of Systemic (Intraperitoneal) Recombinant Crotamine: The Formalin Test

**Figure 7:**
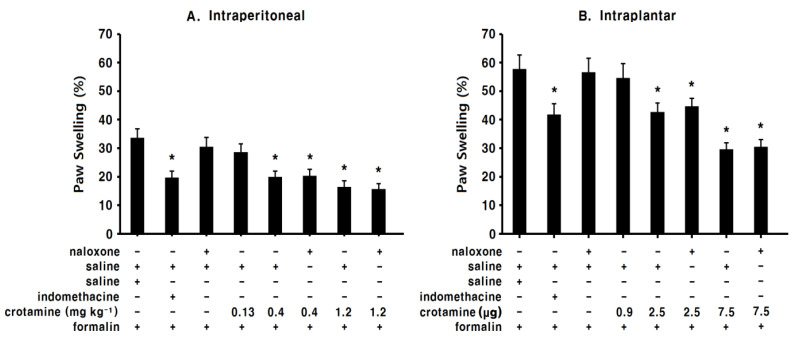
The changes of paw swelling reflecting the anti-inflammatory effects of indomethacin and recombinant crotamine (*n* = 7). (**A**) Indomethacin (10 mg kg^−1^) and recombinant crotamine (0.13–1.2 mg kg^−1^) were intraperitoneally pre-treated 20 min before formalin injection. Naloxone (4 mg kg^−1^) was subcutaneously pre-treated 10 min before recombinant crotamine (0.4 and 1.2 mg kg^−1^) administration. Each value indicates mean ± SEM. * *p* < 0.05 vs. saline-control group, from one-way ANOVA with Tukey’s post hoc test. (**B**) Indomethacin (50 μg 10 μL^−1^) and recombinant crotamine (0.9–7.5 μg 10 μL^−1^) were intraplantarly pre-treated 15 min before formalin injection. Naloxone (5 μg 10 μL^−1^) was intraplantarly pre-treated 15 min before recombinant-crotamine (2.5 and 7.5 μg 10 μL^−1^) administration. Each value indicates mean ± SEM. * *p* < 0.05 vs. saline-control group, from one-way ANOVA with Tukey’s post hoc test.

In the saline-control group, the paw swelling (%) in the left hind paw was 33.6 ± 3.2% (Figure 7A). The paw swelling (%) was significantly suppressed in the intraperitoneal indomethacin (10 mg kg^−1^) group (19.7 ± 2.3%, Figure 7A). In the low-dose intraperitoneal recombinant-crotamine (0.13 mg kg^−1^) group, animals showed a similar degree of paw swelling (28.6 ± 2.9%) compared with the saline-control group (Figure 7A). Contrastingly, in the high-dose intraperitoneal recombinant-crotamine (0.4 and 1.2 mg kg^−1^) groups, intraperitoneal recombinant crotamine caused a dose-dependent suppression of paw swelling (19.9 ± 2.1% and 16.4 ± 2.1%, Figure 7A). The anti-inflammatory effect of intraperitoneal recombinant crotamine (0.4 mg kg^−1^) was similar to the effect of intraperitoneal indomethacin (10 mg kg^−1^, Figure 7A). Naloxone itself did not present any anti-inflammatory effects (Figure 7A); further, anti-inflammatory effects of intraperitoneal recombinant crotamine (0.4 and 1.2 mg kg^−1^) were not affected by the intraperitoneal naloxone pre-treatment (Figure 7A).

### 3.6. The Anti-Inflammatory Effects of Peripheral (Intraplantar) Recombinant Crotamine: The Formalin Test

In the case of intraplantar administration, saline (or naloxone), recombinant crotamine, and formalin were administered to the plantar surface of the left hind paw. Therefore, paw swelling (%) was much higher than that in the case of intraperitoneal administration of saline (or naloxone), and recombinant crotamine (Figure 7B). In the saline-control group, paw swelling (%) demonstrated the maximum measurements 3 h after the subcutaneous administration of formalin to the left hind paw (57.8 ± 4.9%, Figure 7B). In the indomethacin group (intraplantar, 50 μg 10 μL^−1^), paw swelling (%) was significantly suppressed (41.8 ± 3.8%, Figure 7B). In the low-dose intraplantar recombinant-crotamine (0.9 μg 10 μL^−1^) group, paw swelling (%) was similar (54.6 ± 5.1%) to that in the saline-control group (Figure 7B). In the high-dose intraplantar recombinant-crotamine (2.5 and 7.5 μg 10 μL^−1^) groups, paw swelling (%) was significantly suppressed (42.7 ± 3.2% and 29.6 ± 2.3%, *p* < 0.05) compared with that in the saline-control group (Figure 7B). The anti-inflammatory effect of intraplantar recombinant crotamine (2.5 μg 10 μL^−1^) was similar to the effect of intraplantar indomethacin (50 μg 10 μL^−1^) (Figure 7B). Naloxone itself did not present any anti-inflammatory effect; anti-inflammatory effects of intraplantar recombinant crotamine (2.5 and 7.5 μg 10 μL^−1^) were not affected by intraplantar naloxone pre-treatment (Figure 7B).

### 3.7. The Effects of Systemic (Intraperitoneal) Recombinant Crotamine on Serum TNF-α Levels

**Figure 8:**
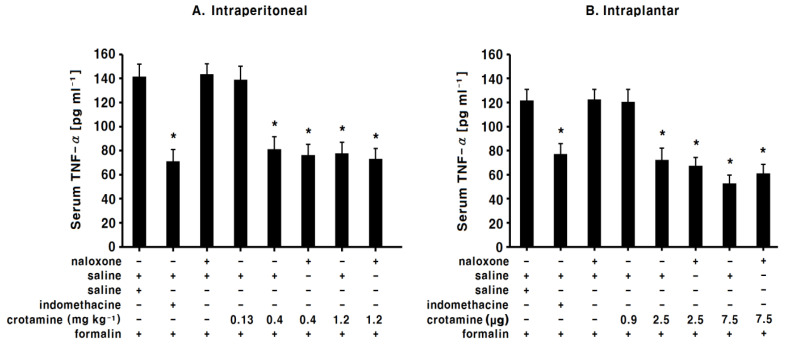
The serum TNF-α levels (*n* = 7). (**A**) In the intraperitoneal indomethacin (10 mg kg^−1^) and recombinant-crotamine (0.4 and 1.2 mg kg^−1^) groups, the mean serum TNF-α concentrations were significantly lower than the saline-control group. The naloxone (4 mg kg^−1^) itself did not present any antinociceptive effect, and the serum TNF-α levels were not affected by the pre-treatment of intraperitoneal naloxone. Each value indicates the mean ± SEM. * *p* < 0.05 vs. saline-control group, from one-way ANOVA with Tukey’s post hoc test. (**B**) In the intraplantar indomethacin (50 μg 10 μL^−1^) and recombinant-crotamine groups (2.5 and 7.5 μg 10 μL^−1^), the mean serum TNF-α concentrations were significantly lower than in the saline-control group. The naloxone (5 μg 10 μL^−1^) itself did not present any antinociceptive effect, and the serum TNF-α levels were not affected by the pre-treatment of intraplantar naloxone. Each value indicates the mean ± SEM. * *p* < 0.05 vs. saline-control group, from one-way ANOVA with Tukey’s post hoc test.

In the saline-control group, serum TNF-α levels were 142.4 (10.8) pg mL^−1^ 3 h after formalin injections (Figure 8A). In the indomethacin group, serum TNF-α levels were significantly decreased (73.6 ± 9.7 pg mL^−1^, *p* < 0.05) compared with that in the saline-control group (*p* < 0.05, Figure 8A). In the low-dose recombinant-crotamine group (0.13 mg kg^−1^), the serum TNF-α levels were similar (134.3 ± 11.9 pg mL^−1^) to those in the saline-control group (Figure 8A). In the high-dose recombinant-crotamine (0.4 and 1.2 mg kg^−1^) groups, serum TNF-α levels were significantly decreased (81.7 ± 7.6 and 77.8 ± 9.7 pg mL^−1^, *p* < 0.05) compared with those in the saline-control group (Figure 8A). Naloxone itself did not present any antinociceptive effect; serum TNF-α levels were not affected by intraperitoneal naloxone pre-treatment (Figure 8A).

### 3.8. The Effects of Peripheral (Intraplantar) Recombinant Crotamine on Serum TNF-α levels

In the saline-control group, serum TNF-α levels were 121.6 ± 9.4 pg mL^−1^ 3 h after formalin injections (Figure 8B). In the indomethacin group, serum TNF-α levels were significantly decreased (77.3 ± 8.6 pg mL^−1^, *p* < 0.05) compared with those in the saline-control group (Figure 8B). In the low-dose recombinant-crotamine (0.9 μg 10 μL^−1^) group, serum TNF-α levels were similar (120.5 ± 10.3 pg mL^−1^) to those in the saline-control group (Figure 8B). In the high-dose recombinant-crotamine (2.5 and 7.5 μg 10 μL^−1^) groups, serum TNF-α levels were significantly decreased (72.3 ± 9.7 and 52.7 ± 7.1 pg mL^−1^, *p* < 0.05) compared with those in the saline-control group (Figure 8B). Naloxone itself did not present any antinociceptive effect; serum TNF-α levels were not affected by the intraplantar naloxone pre-treatment (Figure 8B).

### 3.9. The Effects of Systemic (Intraperitoneal) Recombinant Crotamine on the Rotarod Test

**Figure 9:**
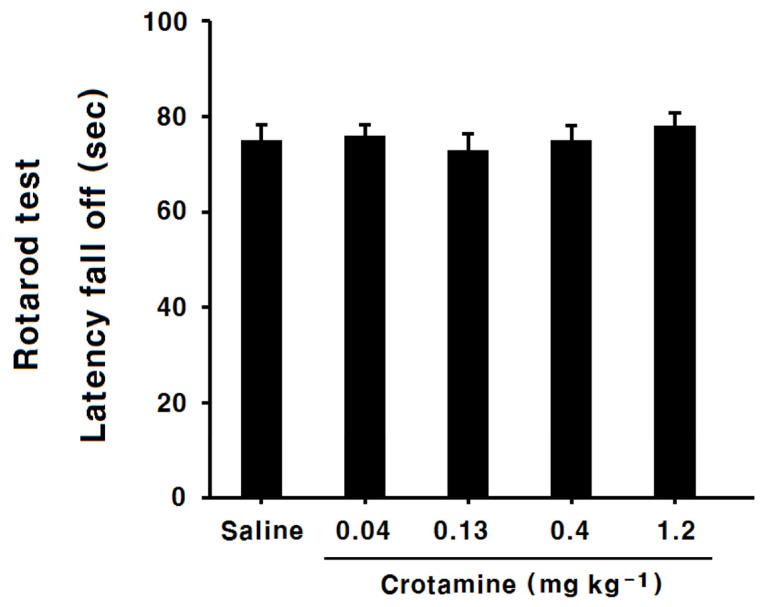
Effect of intraperitoneal recombinant crotamine (0.04–1.2 mg kg^−1^) administration on performance in the rotarod test. Two trials were performed in each group. There were no significant differences among the groups in the measured latency. Each value indicates mean ± SEM.

Higher doses of recombinant crotamine could induce paralysis in mice. To distinguish between paralysis and antinociception in the above assays, the rotarod test was used. In the test, no significant differences were found for the latency time between recombinant-crotamine (0.04, 0.13, 0.4, and 1.2 mg kg^−1^)-injected groups and the saline group (Figure 9). Therefore, we could assume that the observed effects were indeed antinociception.

## 4. Discussion

The present study revealed that intraperitoneal and intraplantar application of recombinant crotamine had antinociceptive effects, which were not influenced by intraperitoneal or intraplantar naloxone pre-treatment. Moreover, naloxone pre-treatment did not affect the anti-inflammatory response of intraperitoneal and intraplantar recombinant crotamine.

### 4.1. The Antinociceptive Effects of Systemic (Intraperitoneal) Recombinant Crotamine: The Hot-Plate Test and the Acetic-Acid-Induced Writhing Method

The hot-plate test is a specific central antinociceptive test [24]. In the present study, intraperitoneally administered recombinant crotamine showed significant and dose-dependent antinociceptive effects in the hot-plate test, suggesting that antinociceptive effects of recombinant crotamine involve central mechanisms. Intraperitoneally administered acetic acid was reported to cause inflammatory pain by inducing capillary permeability [25]. In the present study, writhes by intraperitoneal administration of acetic acid in mice were quantified by abdominal muscle contraction along with a stretching of the hind paw. The number of writhes significantly decreased with intraperitoneal recombinant crotamine prior to the acetic-acid injection in a dose-dependent manner. This suggests that antinociceptive effects of recombinant crotamine involve peripheral mechanisms also. Mancin et al. reported that a 44.5 μg kg^−1^ dose of the native crotamine had an analgesic effect that was 50% of that in the control group in the acetic-acid-induced writhing method [8]. However, in the present study, 0.4 mg kg^−1^ dose of recombinant crotamine had an antinociceptive effect that was 50% of that in the control group. Therefore, the potency of our recombinant crotamine was approximately 10% of the previously reported native crotamine. We do not have an explanation for this difference.

### 4.2. The Antinociceptive Effects of Systemic (Intraperitoneal) or Peripheral (Intraplantar) Recombinant Crotamine: The Formalin Test

The formalin test is the most predictive measure of acute pain and was used as a model for tonic and localized inflammatory pain [26]. Intraplantar injection of formalin causes a local tissue injury to the paw and it evokes characteristic biphasic licking, biting, and flinching responses. Phase 1 (early phase) reflects acute neurogenic pain, and phase 2 (late phase) corresponds to inflammatory pain responses, which are primarily because of the activation of a wide range of neuronal dorsal horns [27]. In the present study, we injected formalin to the plantar surface of the paw, and the characteristic biphasic responses were observed. Pre-treatment of intraperitoneal recombinant crotamine significantly attenuated the phase 2 responses in a dose-dependent manner. Indomethacin was treated before formalin administration as a positive-control group. The nociceptive response was significantly attenuated in phase 2, demonstrating that the antinociceptive potency of recombinant crotamine is similar to that of indomethacin. Indomethacin is an inhibitor of prostaglandin synthesis [28], and it reduces sensitization in the primary afferent neurons and at the spinal cord. Prostaglandins sensitize peripheral nociceptors and enhance the excitability of the nerve fiber to increase inflammation and nociception [29]. It has been suggested that phase 2 (late phase) seems to be an inflammatory response with inflammatory pain and it can be inhibited by anti-inflammatory drugs. These data are in accordance with the idea that drugs act primarily as central analgesics, which inhibit both phase 1 and phase 2. However, peripherally acting drugs are not as effective as central analgesics, and inhibit phase 2 (late phase) more [27].

As shown in Figure 3 and Figure 5, there were no significant differences in AUC among each group in phase 1. However, the mean nociceptive scores at the first 3 min time point but not the 6- and 9-min time points of phase 1 produced significant differences among the groups. Considering phase 1 of the formalin test reflects acute neurogenic pain, the results suggest the possibility of the direct antinociceptive effects of recombinant crotamine on neurons during the early time point.

### 4.3. The Anti-Inflammatory Effects of Systemic (Intraperitoneal) or Peripheral (Intraplantar) Recombinant Crotamine: The Formalin Test

In the present study, we investigated the anti-inflammatory effect of recombinant crotamine by measuring changes in paw volume. Compared with the saline-control group, the dose of intraperitoneal recombinant crotamine was significantly proportional to the suppression of paw swelling in a dose-dependent manner.

In the high-dose groups of intraplantar recombinant crotamine, paw swelling was significantly suppressed in a dose-dependent manner compared to the saline-control group. The anti-inflammatory effect of intraperitoneal recombinant crotamine was similar to the effect of intraperitoneal indomethacin. The anti-inflammatory effect of intraplantar recombinant crotamine was similar to the effect of intraplantar indomethacin. Taken together, these suggest that intraperitoneal and intraplantar recombinant crotamine have an anti-inflammatory effect.

### 4.4. The Effects of Systemic (Intraperitoneal) and Peripheral (Intraplantar) Recombinant Crotamine on Serum TNF-α Levels

In the present study, we measured serum TNF-α levels as a biochemical surrogate marker of inflammation induced by the formalin test in mice. Inflammatory stimuli release reactive oxygen species such as NO and ${O_{2}^{•}}^{-}$ proinflammatory factors such as TNF-α, and pronociceptive mediators such as cytokines [30,31]. TNF-α is a major proinflammatory cytokine, and increased TNF-α is associated with pathological pain [32,33,34]. Compared to the saline-control groups, the higher dose groups of both intraperitoneal and intraplantar recombinant crotamine significantly decreased serum TNF-α levels. Therefore, the antinociceptive effect and anti-inflammatory effect of recombinant crotamine is related to TNF-α.

### 4.5. Recombinant Crotamine and Opioid Receptors

Mancin et al. demonstrated that naloxone counteracted the analgesic effects of intraperitoneally administered crotamine purified from venom [8]. Naloxone is a competitive antagonist of opioid receptors, which has been used clinically for many years to reverse opioid-mediated effects [35,36]. Mancin’s results suggest that crotamine’s analgesic effects are opioid-dependent. However, in the present study, the antinociceptive effect of intraperitoneal and intraplantar recombinant crotamine was not affected by intraperitoneal and intraplantar naloxone pre-treatment. In addition, the anti-inflammatory effect of intraperitoneal and intraplantar recombinant crotamine was not affected by the pre-treatment of the intraperitoneal and intraplantar naloxone. The serum TNF-α levels were not affected by the pre-treatment of the intraperitoneal and intraplantar naloxone. Taken together, these results suggest that purified recombinant crotamine possesses significant antinociceptive and anti-inflammatory effects that do not appear to be related to opioid receptors.

### 4.6. Limitations of This Study

The homemade recombinant crotamine demonstrated significant antinociceptive effects consistent with the results by native crotamine purified from snake venom [8]. However, there are several differences, such as potency of crotamine and inhibition by naloxone. It would be necessary to test the native and recombinant crotamine in the same experimental design simultaneously in the same laboratory conditions in the future. In addition, different concentrations and time intervals of naloxone, acetic acid, and formalin should be tested.

## 5. Conclusions

The present study showed that the purified recombinant crotamine possesses significant antinociceptive and anti-inflammatory effects that do not appear related to opioid receptors. Antinociceptive and anti-inflammatory effects of intraperitoneal and intraplantar recombinant crotamine are related to TNF-α. Recombinant crotamine could be developed as an antinociceptive and anti-inflammatory agent in the central and peripheral sites. Clinically, this study provides a background for the clinical use of recombinant crotamine for treating pain using an opioid-independent pathway.

## Figures and Tables

**Table 1:** Experimental design for the hot-plate test (*n* = 7 in each group).

	Saline	Crotamine (mg/kg)			
		0.04	0.13	0.4	1.2
Saline	Group 1	Group 3	Group 5	Group 7	Group 9
Naloxone	Group 2	Group 4	Group 6	Group 8	Group 10

**Table 2:** Experimental design for the acetic-acid-induced writhing method (*n* = 7 in each group).

	Saline	Crotamine (mg/kg)			
		0.04	0.13	0.4	1.2
Saline	Group 1	Group 3	Group 5	Group 7	Group 9
Naloxone	Group 2	Group 4	Group 6	Group 8	Group 10

**Table 3:** Experimental design for the formalin test for antinociceptive and anti-inflammatory action of systemic (intraperito-neal) recombinant crotamine (*n* = 7 in each group).

	Saline	Indomethacin	Crotamine (mg/kg)		
			0.13	0.4	1.2
Saline	Group 1	Group 2	Group 4	Group 5	Group 7
Naloxone	Group 3			Group 6	Group 8

**Table 4:** Experimental design for the formalin test for antinociceptive and anti-inflammatory action of peripheral (intra-plantar) recombinant crotamine (*n* = 7 in each group).

	Saline	Indomethacin	Crotamine (μg 10 μL^−1^)		
			0.9	2.5	7.5
Saline	Group 1	Group 2	Group 4	Group 5	Group 7
Naloxone	Group 3			Group 6	Group 8

## Data Availability

The datasets generated and analyzed during the present study are available from the corresponding authors on reasonable request.

## Supplementary Materials

The following are available online at https://www.mdpi.com/article/10.3390/toxins13100707/s1, Figure S1: A real time picture of transparent acrylic observation cage.
