# Supplementary material for: Antinociceptive and Anti-Inflammatory Effects of Recombinant Crotamine in Mouse Models of Pain
Source: Toxins (Basel). 2021 Oct 6;13(10):707. doi: 10.3390/toxins13100707 (PMC8538437; doi:10.3390/toxins13100707)
Supplement: Supplementary file 1 [file toxins-13-00707-s001.zip › toxins-1381888 supplimentay .pdf]

Supplementary Information

Takuto Oyama, Motoyasu Miyazaki, Michinobu Yoshimura, Tohru Takata, Hiroyuki Ohjim

and Shiro Jimi

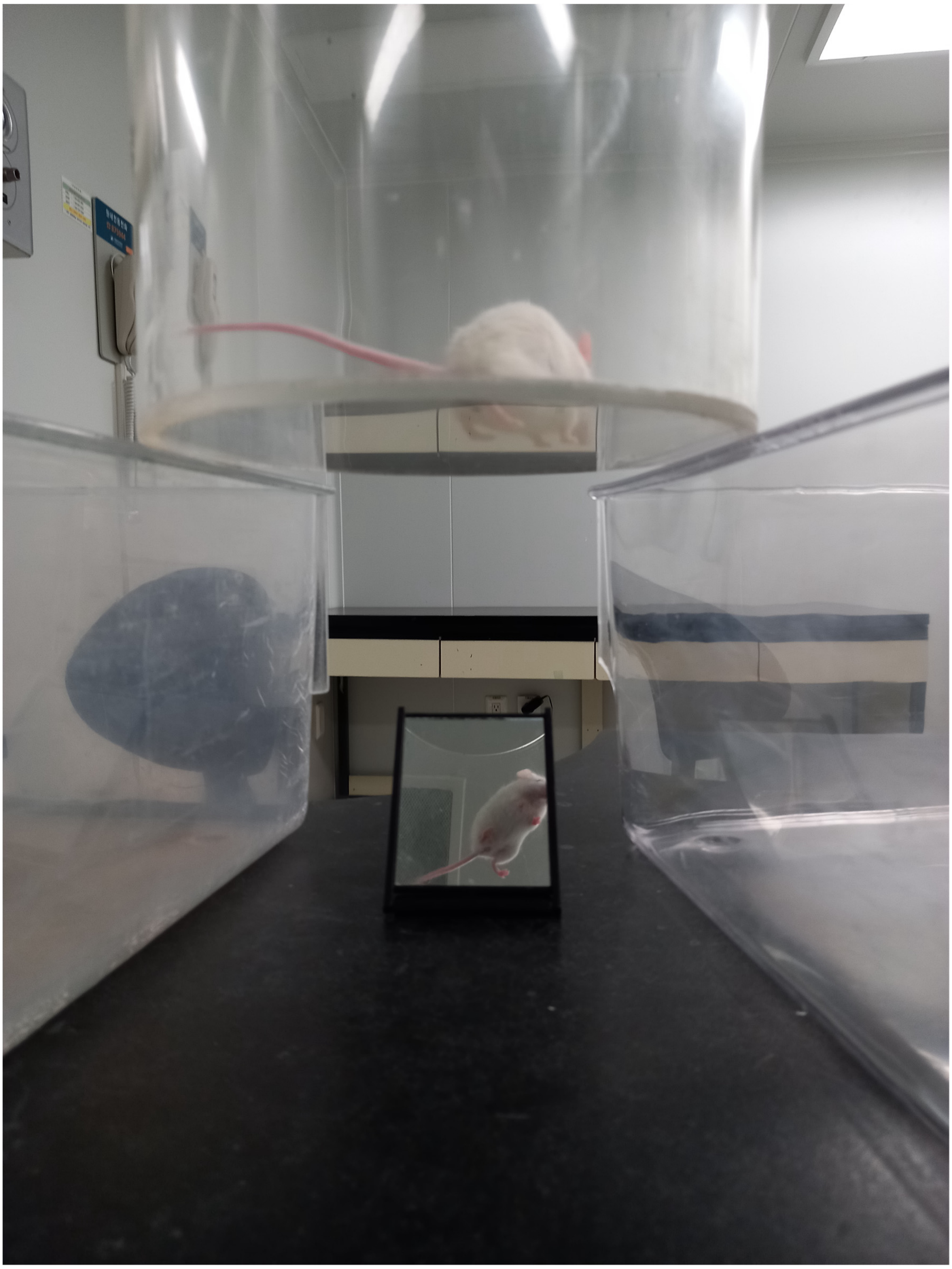

Supplementary Figure S1. A real time picture of transparent acrylic observation cage.
